# Supplementary material for: The causal role of circulating inflammatory markers in osteoporosis: a bidirectional Mendelian randomized study
Source: Front Immunol. 2024 Jul 18;15:1412298. doi: 10.3389/fimmu.2024.1412298 (PMC11291241; doi:10.3389/fimmu.2024.1412298)
Supplement: Supplementary file 1 [file DataSheet_1.zip › supplementary material/CIM-OSTEOPOROSIS /GCST90274783.csv_forest.pdf]

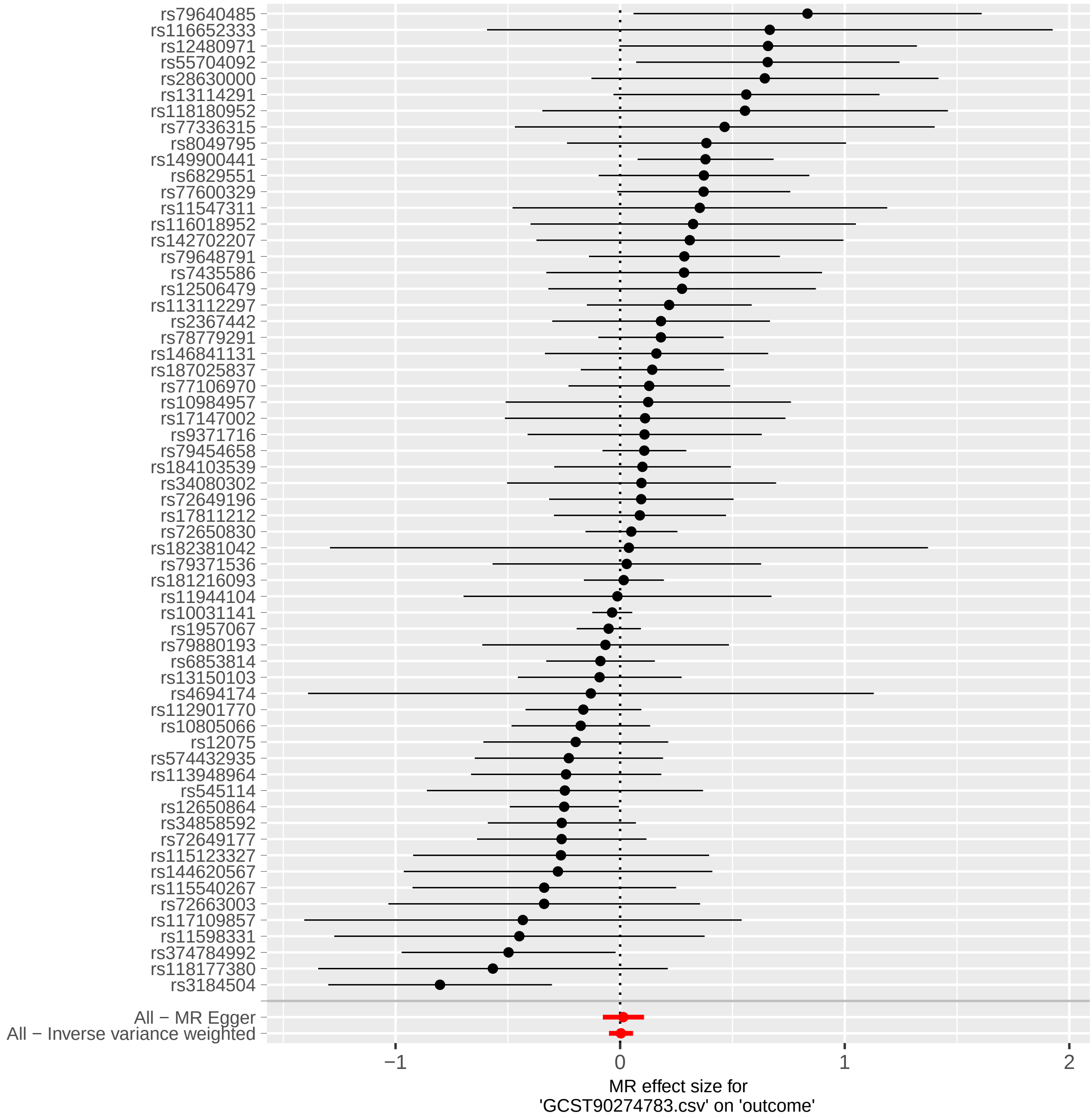
rs79640485
rs116652333
rs12480971
rs55704092
rs28630000
rs13114291
rs118180952
rs77336315
rs8049795
rs149900441
rs6829551
rs77600329
rs11547311
rs116018952
rs142702207
rs79648791
rs7435586
rs12506479
rs113112297
rs2367442
rs78779291
rs146841131
rs187025837
rs77106970
rs10984957
rs17147002
rs9371716
rs79454658
rs184103539
rs34080302
rs72649196
rs17811212
rs72650830
rs182381042
rs79371536
rs181216093
rs11944104
rs10031141
rs1957067
rs79880193
rs6853814
rs13150103
rs4694174
rs112901770
rs10805066
rs12075
rs574432935
rs113948964
rs545114
rs12650864
rs34858592
rs72649177
rs115123327
rs144620567
rs115540267
rs72663003
rs117109857
rs11598331
rs374784992
rs118177380
rs3184504
All − MR Egger
All − Inverse variance weighted
−1
0
1
2
MR effect size for
'GCST90274783.csv' on 'outcome'
